# When wrong is assumed right: a response to vieta

**DOI:** 10.1017/S0033291724002101

**Published:** 2024-09

**Authors:** Cecilia Samamé

**Affiliations:** Universidad Católica del Uruguay, Montevideo, Uruguay

**Keywords:** bipolar disorder, neuroprogression, staging

To the Editor:

I sincerely appreciate the commentary recently published by Professor Vieta (2024), especially because he is a world-renowned leader in the field of bipolar disorders (BDs). However, a number of important concerns arise from his article which I believe warrant consideration and further discussion.

In a peculiar attempt to explain ‘crucial concepts of science’, Vieta states that ‘while hypotheses may be right or wrong, models are better or worse’ and argues that neuroprogression is actually a model: the model that describes the course of cognitive dysfunction in BDs. However, while hypotheses can be proven false, assuming the ‘rightness’ of a hypothesis represents a formal fallacy (affirming the consequent). At best, a scientific hypothesis can survive several opportunities for disproof and, when that occurs, be considered a model, a description of a phenomenon that allows us to make specific predictions. Interestingly, Vieta assumes that neuroprogression has reached the status of a model, that is, an adequately tested hypothesis whose observational consequences have never been disproven. However, as exemplified by his recent commentary, the empirical basis for such an assumption is very weak.

First, Vieta confuses correlation with causality when assuming that the well-documented association between the number of manic episodes and cognitive dysfunction is evidence for an ‘impact of manic episodes’. At the same time, he fails to cite research findings that do not support neuroprogression as an explanation for this correlation. Indeed, baseline cognitive impairment has been shown to be a significant predictor of shorter time to recurrences after adjusting for confounders, thus suggesting that neuropsychological dysfunction might be a severity marker associated with a poorer clinical course rather than being the consequence of the cumulative effect of multiple mood episodes (Martino et al., 2013).

Strikingly, Vieta also argues that the findings of optimal cognitive outcomes during childhood and adolescence in people with subsequent development of BD, which contrast with the deficits observed among diagnosed individuals, is evidence for neuroprogression. However, poor cognitive and functional outcomes have also been reported before illness onset (MacCabe et al., 2010). In addition, it is important to note that studies exploring premorbid functioning used general intelligence tests or proxies for cognitive functioning such as scholastic achievement. Good performance on such measures does not preclude the presence of deficits in specific neurocognitive domains. Furthermore, even if cognitive decline occurs with the onset of the disorder or in a prodromal phase, this does not necessarily mean that BDs follow a progressively deteriorating course. Indeed, evidence from late-life patients (Montejo et al., 2022; Strejilevich & Martino, 2013) and longitudinal studies with long follow-up periods do not support worsening of cognitive outcomes (Samamé, Cattaneo, Richaud, Strejilevich, & Aprahamian, 2022). Within this context, the author proposes a number of ad hoc hypotheses in order to prevent neuroprogression from being falsified (e.g. ‘the most severe and difficult-to-treat patients tend to be lost at follow-up or die’) and such hypotheses cannot be falsified either.

As stated by Vieta, conspicuous cognitive heterogeneity exists within the bipolar spectrum thus reflecting different processes subserving BDs. In the absence of a single bipolar-specific process, the onset and long-term course of these disorders cannot be predicted and, consequently, BDs may not be stageable conditions as is assumed by neuroprogression promoters. Interestingly, Vieta discusses heterogeneity by presenting this issue as a false dilemma between neuroprogression and neurodevelopment, which by no means reflects the view expressed by the author of the current letter (Samamé et al., 2022).

It is important to note that one of the most troubling consequences of accepting neuroprogression is the assumption that resistant BD presentations are late stages of the illness that require more aggressive treatments, including a more intensive exposure to pharmacotherapy which in some cases is associated with cycle acceleration or cognitive impairment. Then, if a patient has increasingly more episodes or worsening of cognitive functioning, this outcome is interpreted as evidence for progression, thus resulting in a self-fulfilling prophecy. It is evident that such concerns no not deny the fact that early diagnosis and treatment are necessary.

Finally, Vieta assumes that functional remediation is a key approach toward improving functional outcomes at different ‘stages’ of BD. At the same time, he discredits the findings of a recent meta-analysis of randomized controlled trials (Samamé, Durante, Cattaneo, Aprahamian, & Strejilevich, 2023) by stating that meta-analyses ‘mix apples with oranges’. However, in this meta-analytic study, effect sizes were quite consistent across trials and subanalyses were performed considering only studies of cognitively or functionally impaired patients, in which no effect of cognitive/functional remediation was observed. Not even in the trial by Vieta's group (Torrent et al., 2013) functional remediation was found to be more efficacious than psychoeducation.

This said, scientific psychiatry does not appear to find its rightful place where logical errors, ad hoc hypotheses, and personal opinions remain recurring elements.
